# Glycogenolysis in Acquired Glioma Resistance to Temozolomide: A Role for the [Ca^2+^]_i_-dependent Activation of Na,K-ATPase/ERK_1/2_ Signaling

**DOI:** 10.3389/fphar.2018.00873

**Published:** 2018-08-07

**Authors:** Junnan Xu, Ye Zhang, Xiangyu Guo, Tao Sun

**Affiliations:** ^1^Department of Medical Oncology, Cancer Hospital of China Medical University, Liaoning Cancer Hospital & Institute, Shenyang, China; ^2^Department of Medical Oncology, Key Laboratory of Liaoning Breast Cancer Research, Shenyang, China; ^3^Department of Neurosurgery, Cancer Hospital of China Medical University, Liaoning Cancer Hospital & Institute, Shenyang, China

**Keywords:** temozolomide, glioma, glycogenolysis, GPBB, Na, K-ATPase

## Abstract

Understanding the mechanistic basis for temozolomide (TMZ)-induced glioma resistance is an important obstacle in developing an effective form of chemotherapy for this type of tumor. Glycogenolysis is known to play an essential role in cellular proliferation and potassium homeostasis and involves the glycogen phosphorylase isoenzyme BB (GPBB). In this investigation, plasma GPBB was correlated with TMZ-resistance. Elevated plasma GPBB concentrations were found to be more frequent in a TMZ-resistant cohort of patients with poor survival rates. TMZ inhibits cell proliferation and induces TMZ resistance by upregulating the expression of O(6)-methylguanine-DNA methyltransferase (MGMT). This process requires glycogenolysis, which was confirmed herein by treatment with 1,4-dideoxy-1,4-imino-D-arabinitol hydrochloride, a glycogenolysis inhibitor and a special GPBB inhibitor. Acute TMZ treatment leads to upregulation of [Ca^2+^]_i_, extracellular-regulated kinase (ERK)_1/2_ phosphorylation, and chronic TMZ treatment leads to upregulation of the expression of Na,K-ATPase, ERK_1/2_, and MGMT protein. Upregulation was abolished for each of these by inhibitors of transient receptor potential channel 1 and the inositol trisphosphate receptor. L-channel [Ca^2+^]_i_ inhibitors and RyR antagonists had no such effect. These results demonstrate that [Ca^2+^]_i_-dependent glycogenolysis participates in acquired glioma TMZ-resistance by upregulating MGMT via a Na,K-ATPase/ERK_1/2_ signaling pathway. GPBB and glycogenolysis may therefore represent novel therapeutic targets for overcoming TMZ-resistant gliomas.

## Introduction

Gliomas are the most common and aggressive tumors of the central nervous system. The most aggressive forms of glioma (grade III and grade IV), based on the World Health Organization’s (WHO) classification system, have limited therapeutic options with poor survival rates (Ostrom et al., 2015; Bush et al., 2017). Despite maximal resection, followed by radiation and concomitant temozolomide (TMZ), patients with grade IV glioma have a median survival of only 12–18 months; those with grade III gliomas have a median survival of 2–5 years (Wang and Jiang, 2013; Patel et al., 2017). TMZ, an oral cytotoxic DNA-alkylating chemotherapeutic, is the standard, conventional drug for patients with high-grade gliomas. The use of TMZ has therapeutic benefit in that it prolonged survival by 2.5 months (14.6 months vs. 12.1 months) in a large phase 3 clinical trial, with a twofold improvement in 2-year survival (10.4% vs. 26.1%) (Stupp et al., 2005). Interestingly, an additional study demonstrated that patients with low O(6)-methylguanine-DNA methyltransferase (MGMT) expression, or MGMT promoter methylation, had a significant overall improvement of 46% in survival at 2 years (Happold et al., 2018). Conversely, glioma patients with high expression of MGMT, and reduced MGMT methylation, had a poor response to TMZ. However, there are many limitations to the use of TMZ in patients with high-grade glioma. Multiple genetic and tumor microenvironments suggest that the current use of TMZ is inadequate and does not provide effective and complete protection from high-grade glioma (Altieri et al., 2014; Pan et al., 2015). Furthermore, the mechanistic basis for TMZ resistance is not well understood. Because of the poor survival rate of high-grade glioma patients, it is very important to identify new biomarkers that predict TMZ efficacy and to identify better and more effective treatment strategies.

Increased glucose uptake and enhanced glycolytic rates are found in most malignancies, with abrogation of metabolism for tumor cell growth. Glycogenolysis is an astrocyte-specific process (Gibbs et al., 2006), with abrogation of metabolism for the formation of transmitter glutamate and potassium homeostasis in the brain (Xu et al., 2013; Xu et al., 2014; Gibbs, 2016), the key factor participating in this pathway is Na,K-ATPase. Importantly, we found that the studies suggested that inhibition of Na,K-ATPase triggers hybrid cell death and serves as an underlying mechanism for an enhanced TMZ effect on glioblastoma cells (Chen et al., 2014) and as a blockade of the calcium-activated potassium channel KCa3.1, and with TRAM-34 has co-adjuvant effects with TMZ, reducing GL261 glioma cell migration, invasion and colony-forming activity, increasing apoptosis, and forcing cells to pass the G2/M cell cycle phase, likely through cdc2 de-phosphorylation (D’Alessandro et al., 2016). Mibefradil (MIB), a selective T-type calcium channel blocker, has preclinical and clinical activity in high-grade gliomas (HGGs) with a comprehensive characterization of Cav3.2 in this mechanism (Holdhoff et al., 2017; Zhang et al., 2017). Based on our previous study and the literatures that led us to perform our initial experiment, interestingly, these results demonstrate median plasma Glycogen phosphorylase isoenzyme BB (GPBB) concentration to be significantly higher in high-grade glioma patients with TMZ resistance than in TMZ-sensitive patients. GPBB is activated during ischemia and tumor development, increasing glycogen degradation with increased detection in the blood. Several studies have demonstrated plasma GPBB to be correlated with myocardial ischemia and preterm preeclampsia (Horacek et al., 2013; McCarthy et al., 2016). Glycogen synthase kinase (GSK)3beta promotes glycogen synthase and the reduction of glycogenolysis in brain and glioma cells (Luo, 2009). The way in which GSK3beta is regulated differs in response to chemotherapy and is dependent upon tumor cell type. For example, GSK3beta functions as a tumor promoter for colon cancer or ovarian tumor cells and therefore provides resistance to chemotherapy. However, GSK3beta acts as a tumor suppressor and its activation sensitizes tumor cells to chemotherapy in breast cancer (Luo, 2009). Pyko et al. (2013) demonstrated that GSK3beta inhibiting drugs reduce glycogen synthesis and eliminate the suppressive effect of GSK3beta on TMZ resistance by the recruitment of DNA methyltransferase (DNMT)3A and by reduction of MGMT expression, and that this finally ultimately sensitizes glioblastoma cells to TMZ. However, a relationship between glycogen content (synthetic and degradative) and sensitivity to glioma chemotherapy has not yet been fully established. Glycogenolysis is intracelluar free calcium ion [Ca^2+^]_i_-dependent, not only in muscle, but also in brain slices (Ververken et al., 1982; Ozawa, 2011). An intense K^+^ uptake, with glycogenolysis activation, is triggered by different extracellular K^+^ concentrations due to activation of the Na^+^, K^+^, Cl^-^, and water transporter NKCC1 and Na,K-ATPase, which is secondary to depolarization mediated by the opening of L-channels or transient receptor potential channel 1 (TRPC1) for [Ca^2+^]_i_ release (Yan et al., 2013). Therefore, we considered that glycogenolysis (via calcium-associated pathways) would play a role in cAMP-stimulation and the therapeutic effects of TMZ.

Notably, our previous small-cohort study suggested that high expression of GPBB in tissues of glioma patients was correlated with poor overall survival (OS, unpublished data). The present study is based on a discussion by Professor Hertz that glioma GPBB upregulation, in response to TMZ, may require glycogenolysis and its associated pathways. It is important to note that roles for GPBB and glycogenolysis in glioma and TMZ resistance remain unclear.

In the present study, plasma GPBB from high-grade glioma patients was examined in order to determine if plasma GPBB correlated with TMZ sensitivity and OS. We assessed this by measuring free [Ca^2+^]_i_ release and cell proliferation in the presence and absence of inhibitors of potential signaling pathways, with and without stimulation (cAMP or TMZ). Chronic TMZ treatment was examined for its effect on MGMT as well as an analysis of the effects of canrenone, a specific inhibitor of Na,K-ATPase that is upstream of mitogen-activated protein kinase (MAPK) and increases [Ca^2+^]_i_ (Müller et al., 2014). In order to assess potential signal pathways, the expression of Na,K-ATPase and ERK_1/2_ was assessed during chronic TMZ treatment; the phosphorylation of ERK_1/2_ and AKT was assessed during acute TMZ treatment.

## Materials and Methods

### Reagents

Chemicals used in the preparation of media, and most other chemicals, including DAB (1,4-dideoxy-1,4-imino-D-arabinitol hydrochloride), cAMP (cyclic adenosine monophosphate), temozolomide, xestospongin C (1R,4aR,11R,12aS,13S,16aS,23R,24aS)-eicocahydro-5H,17H-1,23:11,13-diethano-2H,4H-[1,11], dioxacycloeicosino [2,3-b:12,13-b1] dipyridine, nifedipine, and canrenone were purchased from Sigma (St. Louis, MO, United States). TRPC1 channel antibody (SC-15055) was obtained from Santa Cruz Biotechnology (sc-15055 from Santa Cruz, CA, United States). Oligofectamine^TM^ Reagent for RNA interference, Opti-MEMI, Ryanodine, and U0126 [1,4-diamino-2,3-dicyano-1,4-bis (2-aminophenylthio) butadiene] were purchased from Calbiochem (La Jolla, CA, United States). Fura-2 AM was obtained from Invitrogen (Carlsbad, CA, United States).

### Ethics Statement

This study was approved by the Medical Ethical Committee of Liaoning Cancer Hospital and Institute (No. 20150309-2). Informed consent was obtained from all patients and controls before enrollment in this study. All patients underwent surgery and subsequent TMZ chemotherapy, and all patients underwent a minimum of 3 years of follow-up. The diagnosis was verified by pathological analysis and classified according to the WHO’s standards.

### Cell Culture

The human glioma cell line U251 was purchased from the American Type Culture Collection (ATCC, Rockville, MD). Cell cultures were maintained in Roswell Park Memorial Institute (RPMI) 1640 medium supplemented with 10% fetal bovine serum (FBS) and cultured at 37°C in a humidified atmosphere containing 5% CO_2_ as described in previous study (Zhao et al., 2017).

### Enzyme-Linked Immunosorbent Assay (ELISA) for GPBB

Blood samples, collected in EDTA tubes, were centrifuged at 1,000 ×*g* for 15 min at 4°C. Plasma samples were then kept at -80°C until assayed. Plasma GPBB concentration was measured with GPBB ELISA Kits (CSB-E08475h, CUSABIO, Wuhan, China), according to the manufacturer’s instructions. Readings were made at 450 nm excitation and 570 nm emission with a microplate reader (Sunrise, TECAN, Switzerland). A standard curve was used to assess intensity at different GPBB concentrations from which GPBB concentration was calculated.

### Collagen Gel Droplet Embedded Culture Drug Sensitivity Test (CD-DST)

The CD-DST was used to evaluate the sensitivity of cancer tissue to temozolomide (TMZ) (Lv et al., 2016; Sakuma et al., 2016). In brief, 5-mm cubes of glioma specimens were obtained by surgery and minced with a surgical knife, digested with collagenase, and the dispersed cancer cells incubated in a flask coated with collagen gel. Only viable cells that adhered to the collagen gel layer were collected and added to a reconstructed type I collagen solution (Cellmatrix Type CD; Kurabo Industries, Ltd., Osaka, Japan). TMZ (5 μM) was added to each well of 6-well plates. The wells were incubated for 24 h at 37°C. Subsequent to the removal of the medium containing TMZ, each well was incubated with prepared culture media **(**PCM)-2 medium (Kurabo Industries, Ltd.,) for 7 days. Neutral red was then added to stain colonies in collagen gel droplets, which were then fixed with formalin. Images of the stained gels were acquired with a video microscope (VH-5910; Keyence, Osaka, Japan) and cell proliferation rates were determined by measuring optical densities. *In vitro* chemosensitivity was expressed as a ratio of the total colony volume of the treated group (T) to that of the control group (C) (T/C ratio). A T/C ratio of ≤ 60% was regarded as TMZ sensitive, with TMZ resistance defined as a T/C ratio of > 60%.

### DNA Pyro-Sequencing for Isocitrate Dehydrogenase (IDH)1/2 Mutations

Pyro-sequencing was performed as previously described (Zeng et al., 2015). Briefly, genomic DNA was extracted from frozen tissues with a QIAamp DNA Mini Kit (Qiagen) according to the manufacturer’s protocol. DNA concentration and quality were then measured using a Nano-Drop ND-1000 Spectrophotometer (NanoDrop Technologies, Houston, TX, United States). Pyrosequencing of IDH1/2 mutations was carried out by Gene-tech (Shanghai, China) and performed on a Pyro-Mark Q96 ID System (Qiagen, Valencia, CA, United States). The primers 5′-GCT TGT GAG TGG ATG GGT AAA AC-3′ and 5′-Biotin-TTG CCA ACA TGA CTT ACT TGA TC- 3′ for IDH1 were used for PCR amplification, and the primer 5′-TGG ATG GGT AAA ACC T-3′ for IDH1 was used for sequencing.

### Methylation-Specific Polymerase Chain Reaction (PCR) for the MGMT Promoter

The MGMT methylation was detected using methylation-specific PCR (MSP), as described in previous study (Fiano et al., 2014). Genomic DNA from each sample (2 μg) was treated with sodium bisulfite using the Epitect Bisulfite Kit (Qiagen Valencia, CA, United States) performed on a Pyro-Mark Q96 ID System (Qiagen, Valencia, CA, United States). The primer sequences for the methylated reaction were 5′-TTT CGA CGT TCG TAG GTT TTC GC-3′ and 5′-GCA CTC TTC CGA AAA CGA AAC G-3′, and those for the non-methylated reaction were 5′-TTT GTG TTT TGA TGT TTG TAG GTT TTT GT-3′ and 5′-AAC TCC ACA CTC TTC CAA AAA CAA AAC A-3′.

### 1p/19q Co-deletion Analysis by Fluorescence *in situ* Hybridization

Fluorescence *in situ* hybridization (FISH) was performed for 1p/19q co-deletion analysis. Control and detection probes were developed from plasmids D1Z1 (1q12) and D1Z2 (1p36.3) for the chromosome 1 study and from bacterial artificial chromosomes (BACs) RP11-413 M18 (19q13) and CTZ-2571 L23 (19q13.3) for the chromosome 19 study. Dual-colored probes against chromosomes 1p and 19q were used to detect chromosomal loss at these loci. A single fluorescent signal in the nucleus was interpreted as chromosomal-arm loss if, in addition, two signals were detected for the control probe.

### Determination of Glycogenolysis

Glycogenolysis determination protocols have been described previously (Xu et al., 2014). Briefly, a suspension of cultured cells was used to measure non-hydrolyzed glycosyl units of glycogen. Two 50 μl aliquots were sampled. The first and second aliquots were added to 150 μl of acetate buffer (0.1 M, pH 4.65) with or without 1% amyloglucosidase (10 mg/ml) at room temperature for 30 min. The fluorescence of reduced nicotinamide adenine dinucleotide phosphate (NADPH), formed in amounts equivalent to glucose metabolized by hexokinase, was then assessed (excitation 340 nm; emission 450 nm). Determination of the difference between the two aliquots provided a measure of the amount of glycogen. Glycogen content was then calculated as the difference between the two aliquots and reported as a molecular weight of 162 g/mol per glycosyl unit of glycogen. The experiments were performed from three to five individual cultures of each treatment.

### MTT Assay

The proliferation potential of U251 glioma cells was determined by MTT assay [3-(4,5-dimethylthiazol-2-yl)-2,5-diphenyltetrazolium bromide]. Briefly, cells were seeded into 96-well plates (BD Biosciences, San Jose, United States) at a cell density of 1 × 10^3^ cells/well in growth medium. Cell growth was then assayed by the addition of 20 μL of MTT (5 mg/mL; Sigma–Aldrich) to each well, and the plate was incubated at 37°C for 4 h. The proliferation assay was performed for 3 days, and cell growth was assayed every 24 h. The reaction was stopped by the addition of 200 μL dimethyl sulfoxide (Sigma–Aldrich). Optical density was measured at 490 nm with a microplate reader (Sunrise, TECAN, Switzerland). The experiments were performed from three to five individual cultures of each treatment.

### Determination of [Ca^2+^]_i_

The [Ca^2+^]_i_ determination protocols have been described previously (Yan et al., 2013). Briefly, in order to determine [Ca^2+^]_i_, an Olympus IX71 live cell imaging fluorescence microscope (Tokyo, Japan) was used to record the fluorescence intensity of fura-2 AM in saline solution (NaCl 137 mM; KCl 5.4 mM; KH_2_PO_4_ 0.44 mM; NaHCO_3_ 4 mM; CaCl_2_ 1.3 mM; MgSO_4_ 0.8 mM; MgCl_2_ 5 mM, and glucose 10 mM, pH 7.4) for 30 min at 37°C for glioma cells grown on coverslips coated with polylysine. After two washes with this saline solution, coverslips were either perfused in the saline solution (control), or TMZ was carefully added to the saline at time zero. Readings were taken at 340 and 380 nm excitation and 510 nm emission at 20-s intervals. The incubation continued for 22 min. Between 13 and 20 cells were selected for each coverslip, and three to five coverslips were assessed in each experimental group. The represents of this experiment performed means from 38 to 81 cells on three to five individual coverslips of each treatment.

### Western Blot Analysis

The U251 cells were lysed and the concentration of protein quantified using a BSA kit (cat no. QJ223202; Thermo Fisher Scientific, Inc.). In Brief as described in previous study (Liang et al., 2014), an equal amount (50 mg/lane) of protein lysate was resolved in a 10% sodium dodecyl sulfate polyacrylamide gel electrophoresis (SDS–PAGE) gel. After transfer to nitrocellulose (NC) membranes, the NC membranes were incubated with the first antibody, specific to phosphorylated ERK_1/2_ (1:3000), phosphorylated AKT (1:1500), Na,K-ATPase (1:1000), AKT (1:1000), MGMT (1:1000), or β-actin (1:3000) for 1.5 h at room temperature. Specific horseradish peroxidase-conjugated secondary antibodies, mouse-anti-rabbit (1:3000), goat-anti-mouse (1:3000), or goat-anti-rabbit (1:5000), were then incubated with the membranes for 2 h and positive staining visualized with an enhanced chemoluminescence (ECL) reagent. The ratios of NKA (Na,K-ATPase) and glyceraldehyde 3-phosphate dehydrogenase (GAPDH) were calculated and ImageJ software (National Institutes of Health, Bethesda, MD, United States) was used to quantify band density. The experiments were performed from three to five individual cultures of each treatment.

**Table 1:** Clinical characteristics of high-grade glioma patients with TMZ sensitivity and TMZ resistance.

	TMZ sensitivity (*n* = 34)	TMZ resistance (*n* = 29)	χ*^2^*	*P*
**Age**				
≥ 60	18	11	1.419	0.233
< 60	16	18		
**Sex**				
Male	13	16	1.807	0.179
Female	21	13		
**Giloma grade**				
III	15	9	1.136	0.287
IV	19	20		
**1p/19q co-deletion**				
Yes	16	7	3.547	0.060
No	18	22		
**IDH1 mutant**				
Yes	7	7	0.114	0.736
No	27	22		
**MGMT methylation**				
Yes	18	16	0.031	0.859
No	16	13		
**Plasma GPBB**			*T*-value	
Mean ± SE	21.42 ± 1.25	31.78 ± 1.30	-5.510	< 0.001


### Statistics Analysis

Statistical analysis was performed using SPSS (version 20.0, IBM Corporation, Armonk, NY, United States) and figures prepared using GraphPad Prism 5. Data are presented as mean ± SEM Statistical differences between individual groups were analyzed by one-way analysis of variance (ANOVA) followed by Fisher’s LSD test. The level of significance was set at *P* < 0.05. Differences between two independent groups were evaluated using a Student-Newman-Keuls test. The cutoff value for GPBB concentration was determined using receiver operating characteristic (ROC) curve analysis for TMZ resistance. OS curves were constructed using the Kaplan–Meier method, and a log-rank test was used to perform comparisons between TMZ sensitivity and TMZ resistance. OS time was calculated from the initiation of treatment to mortality. Individuals who were alive at the time of last follow-up were censored.

## Results

### Elevated Plasma GPBB Concentration in Patients With TMZ Resistance

To investigate the potential role of GPBB in TMZ resistance in patients with high-grade glioma, we enrolled 63 patients with high-grade glioma from the Cancer Hospital of China Medical University. The inhibition ratios (T/C ratio) of surgical primary glioma cells were analyzed by the CD-DST. Results demonstrated that the mean T/C ratio of TMZ was 61.17% (range: 24.75–94.52%). TMZ resistance was defined as an inhibition ratio (T/C ratio) of more than 60%. If the T/C ratio was less than 60%, the case was defined as TMZ sensitive. In this study, 29 cases were TMZ resistant and 34 cases were TMZ sensitive. **Table 1** shows the demographics and clinical characteristics of the patients whose tumors were TMZ resistant and TMZ sensitive. Patients with TMZ resistance were younger than those with TMZ sensitivity, but the difference did not reach statistical significance (*P* = 0.233). Gender, grade, and glioma pathology were not significantly different.

**FIGURE 1:**
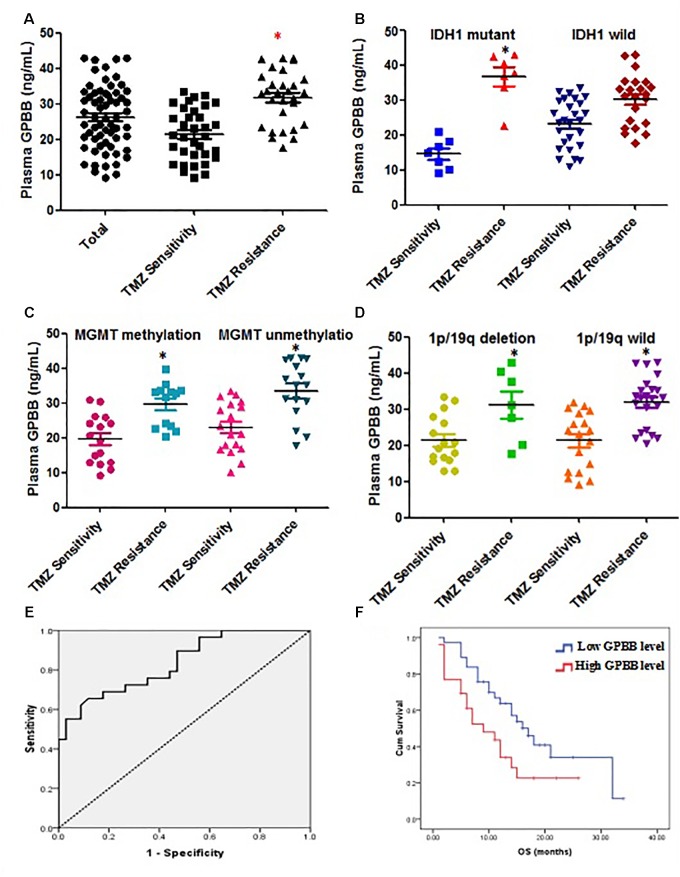
Elevated plasma glycogen phosphorylase BB (GPBB) concentrations in high-grade glioma patients with TMZ resistance compared to those with TMZ sensitivity. Plasma GPBB concentration was measured before surgery. **(A)** Plasma GPBB concentration *in toto*, TMZ-sensitive and TMZ-resistant cohorts. **(B)** Plasma GPBB concentration in IDH1 mutant and IDH1 wild cohorts. **(C)** Plasma GPBB concentration in MGMT methylation and MGMT non-methylation cohorts. **(D)** Plasma GPBB concentration in 1p/19q co-deletion and 1p/19q wild cohorts. **(E)** ROC curve analysis of plasma GPBB concentration in response to TMZ. All values are expressed as means ± SEM, indicated by vertical bars. ^∗^Indicates a statistically significant (*P* < 0.05) difference from TMZ-sensitivity group. **(F)** Survival curve showing overall survival (OS) for high-grade glioma patients with high levels or low levels of plasma GPBB.

**Figure 1A** illustrates the median GPBB plasma concentration. GPBB was consistently and significantly increased in high-grade glioma patients with TMZ resistance (*n* = 34) when compared to TMZ sensitive patients (*n* = 29) (mean 31.78 ng/ml, range 9.17–33.54 ng/ml *vs.* mean 21.42 ng/ml, range 17.61–42.96 ng/ml, *P* < 0.001). This difference remained significant in the IDH 1 mutant cohort (mean 14.62 ng/ml vs. 36.65 ng/ml, **Figure 1B**). However, in the wild type IDH 1 cohort, the results demonstrated no significant differences in the plasma GPBB concentration between TMZ resistant and sensitive groups (mean 23.19 ng/ml *vs.* 30.23 ng/ml, **Figure 1B**). **Figure 1A** shows the mean GPBB plasma concentration to be dramatically higher in patients with TMZ resistance compared to TMZ sensitivity, regardless of 1p/19q co-deletion and MGMT methylation status (MGMT methylation, mean 21.46 ng/ml vs. mean 31.08 ng/ml, *P* < 0.001; MGMT non-methylation, mean 21.39 ng/ml vs. mean 32.00 ng/ml, *P* < 0.001, **Figure 1C**; 1p/19q co-deletion, mean 21.46 ng/ml vs. mean 31.08 ng/ml, *P* < 0.001; 1p/19q wild type, mean 21.39 ng/ml vs. mean 32.00 ng/ml, *P* < 0.001, **Figure 1D**). Overall, these results demonstrate that plasma GPBB is increased in high-grade glioma patients with TMZ resistance.

The ROC curve analysis of plasma GPBB concentration in patients with high-grade glioma was performed to evaluate predictive TMZ sensitivity. Plasma GPBB concentration was shown to have 66% sensitivity and 88% specificity (AUC:0.835, 95% CI 0.737–0.933, **Figure 1E**) in discriminating the response to TMZ *in vitro*. Among all the plasma GPBB concentrations, the sensitivity and specificity of plasma GPBB concentration was maximal at the optimal cut-off value of 30.71. For the group, as the cut-off value of plasma GPBB concentration was increased from 30.71 to 31.04, the sensitivity increased (66–62%) while the specificity decreased (88–91%).

**Figure 1F** demonstrates that patients with high plasma GPBB concentration (>30.71) had a poorer OS than those with low plasma GPBB concentration (7 months vs. 14 months, *P* = 0.032). **Figure 1F** demonstrates agreement between the *in vitro* test results and patient survival.

### Glycogenolysis Participates in TMZ Resistance *in vitro*

**FIGURE 2:**
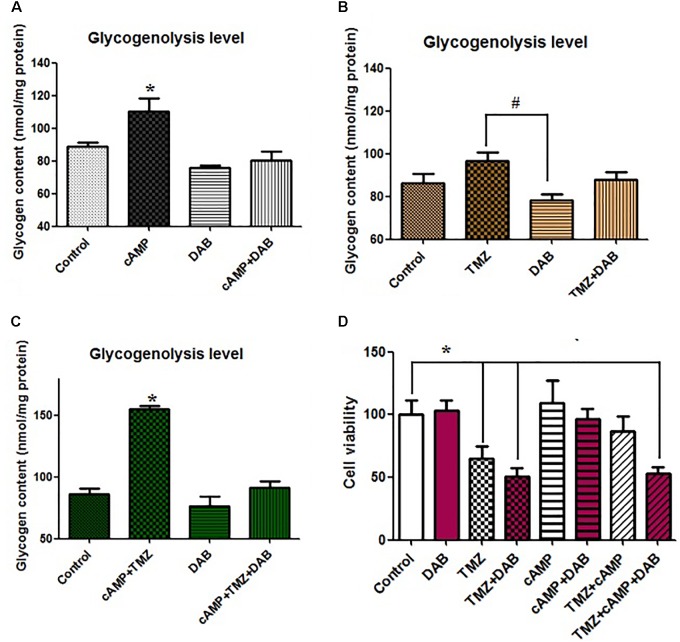
Effect of cAMP and TMZ on glycogenolysis indicated by a reduction in the glycogen content of U251 cells. **(A)** Cultures were incubated in DMEM (containing 7.5 mM glucose) for 20 min without any addition (Cont) or in the presence of 3 μM cAMP, with or without 10 mM DAB, an inhibitor of glycogenolysis. **(B)** Cultures were incubated in DMEM for 20 min under control conditions (Cont) or in the presence of 5 μM TMZ, with or without 10 mM DAB, an inhibitor of glycogenolysis. **(C)** Cultures were incubated in DMEM for 20 min under control conditions (Cont) or in the presence of 3 μM cAMP and 5 μM TMZ, with or without 10 mM DAB, an inhibitor of glycogenolysis. After the experiment, glycogenolysis was determined by measuring glucose content fluorometrically before and after the breakdown of glycogen remaining in the cells. Mean glycogen contents are indicated as calculated glycogen. Reduced glycogen content indicates high activation levels of glycogenolysis. All values are expressed as means ± SEM, indicated by vertical bars, and are from three to five individual cultures. **(D)** Cells were incubated for 20 min, and MTT analysis was performed in the absence of any drug (Control) or in the presence of 5 μM TMZ and/or 3 μM cAMP, with or without 10 mM DAB, an inhibitor of glycogenolysis. ^∗^Indicates a statistically significant (*P* < 0.05) difference from negative controls while # indicates a statistically significant (*P* < 0.05) difference from TMZ.

Remarkably different clinical outcomes have been reported regarding plasma GPBB concentration. GPBB appears to be a key enzyme that enhances glycogenolysis. To better understand the molecular basis for glycogenolysis-associated TMZ resistance, we established high glycogenolysis levels by the infusion of cAMP into U251 cells. The infusion of cAMP (3 μM) into U251 glioma cells stimulated glycogenolysis, **Figure 2A** (*P* < 0.05), while with the control condition, the mean glycogen content was 90 ± 8.1 nmol/mg protein. cAMP dramatically increased glycogenolysis in U251 cells. In spite of the presence of 10 mM glucose in all media, the cAMP increase in glycogenolysis was virtually abolished by the addition of DAB, a glycogenolysis inhibitor and a GPBB inhibitor, which had no effect on control cells. We next investigated whether TMZ in glioma cells directly affected glycogen metabolism and glycogenolysis. As shown in **Figure 2B**, compared to the control, acute TMZ stimulation of U251 cells resulted in somewhat less glycogen degradation than the addition of cAMP. TMZ-induced glycogenolysis was completely abolished with DAB, acting as a GPBB inhibitor. Treatment with TMZ 1 h before cAMP infusion, influenced the stimulatory effect of cAMP on glycogen catabolism by increasing the rate of glycogenolysis, which was reduced to 90 nmol/mg protein by DAB (**Figure 2C**). There was a marked increase in glycogenolysis that was influenced slightly by TMZ.

Furthermore, we investigated the roles of glycogenolysis and GPBB inhibition in TMZ-resistance with regards to cell proliferation and cytotoxicity. Using the MTT assay, *in vitro* cAMP-induced cell glycogenolysis was less vulnerable to TMZ treatment. Compared to the control, cAMP slightly increased U251 cell proliferation via the upregulation of glycogenolysis, which was completely abolished by DAB, **Figure 2D**. In contrast, TMZ was sufficient for a U251 cytotoxic effect and synergized with DAB to inhibit cell proliferation, without changing the cytotoxic effect. cAMP also substantially inhibited TMZ-induced apoptosis. Moreover, the administration of DAB 4 h before cAMP perfusion completely abolished the inhibitory effect of cAMP on TMZ-induced cytotoxicity. TMZ-induced apoptosis was slightly enhanced in U251 cells following pretreatment with DAB. Interestingly, TMZ inhibited U251 cell proliferation and partially promoted cell proliferation and induced TMZ resistance by the upregulation of glycogenolysis. This TMZ effect was enhanced by DAB, which suggests that TMZ resistance requires glycogenolysis.

### Effects on [Ca^2+^]_i_ by Addition of TMZ and/or DAB: A Mechanistic Viewpoint

**FIGURE 3:**
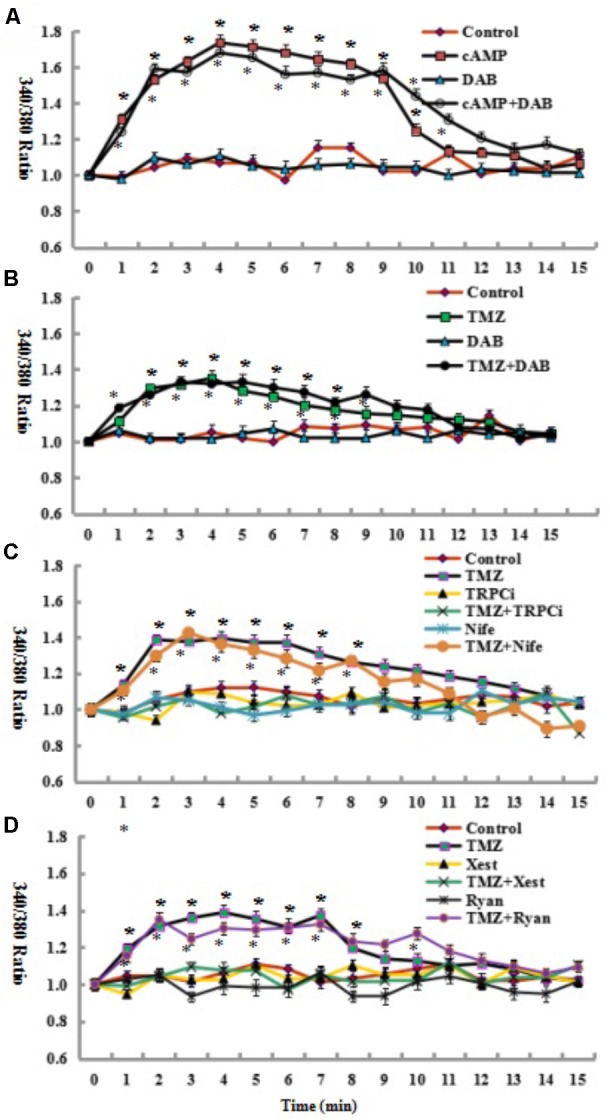
Glycogenolysis is not required for the increase in [Ca^2+^]_i_ induced by the addition of 3 μM cAMP or 5 μM TMZ. **(A)** Following the incubation of fura-2-loaded cells in saline solution for 2 min and a subsequent washing step, the cells were perfused either in a similar solution or in a solution to which an additional 3 μM cAMP had been added at zero time. In some experiments, 10 mM DAB, an inhibitor of glycogenolysis, was added 2 min before the addition of cAMP. All results (340/380 fluorescence ratio of fura-2) after the addition of cAMP and cAMP plus DAB were significantly different (*P* < 0.05) from control conditions. **(B)** An increase in [Ca^2+^]_i_ by 5 μM TMZ added at zero time was similarly unaffected by 10 mM DAB, added as in **(A)**. All results after the addition of TMZ and TMZ plus DAB were significantly different (*P* < 0.05) from control conditions. **(C,D)** An increase in [Ca^2+^]_i_ by 5 μM TMZ added at zero time was prohibited by the addition of 2 μg/mL TRPC1 neutralizing antibody (TRPCi) and 1 μM Xestspongin C (Xest), but not inhibited by 1 μM Nifedipine (Nife), or 1 μM Ryanodine (Ryan), added as in **(A)**. All results after the addition of TMZ, TMZ plus Nife, and TMZ Plus Ryan were significantly different (*P* < 0.05) from control conditions. Results represent means from 38 to 81 cells on three to five individual coverslips. SEM values are indicated by vertical bars. ^∗^Indicates a statistically significant (*P* < 0.05) difference from the drug-free group at the same time point.

To identify whether glycogenolysis is inhibited by the blockade of intracellular calcium ([Ca^2+^]_i_)-mediated signaling pathways, the effect of TMZ on [Ca^2+^]_i_ was assessed. [Ca^2+^]_i_ (as indicated by 340/380 nm fluorescence ratio) was significantly increased by the addition of cAMP, with the increased [Ca^2+^]_i_ rapidly returning to resting values over a period of 15–20 min (**Figure 3A**). As shown in **Figure 3B**, TMZ had a similar effect on [Ca^2+^]_i_, but the release of [Ca^2+^]_i_ was less than with cAMP. Under control conditions, DAB had no effect on [Ca^2+^]_i_ induction by TMZ or cAMP (**Figures 3A,B**). Overall, [Ca^2+^]_i_ was not influenced by the glycogenolysis inhibitor DAB. These results suggest that glycogenolysis occurs downstream of the TMZ/cAMP-induced increase in [Ca^2+^]_i_.

The mechanism underlying the TMZ stimulation of glycogenolysis was assessed by analysis of the effect of [Ca^2+^]_i_-related signal pathway inhibitors on target channels and receptors of the cell membrane and endoplasmic reticulum (ER). As shown in **Figure 3C**, inhibition by a neutralizing antibody of the transient receptor potential channel 1 (TRPC1), an ER-activated calcium channel in the TRPC1 plus TMZ group, resulted in a significant reduction in [Ca^2+^]_i_ release by TMZ. There was no difference between control cells and cells treated with TRPC1 neutralizing antibody plus TMZ. However, when U251 cells were administered with nifedipine (**Figure 3C**), a dihydropyridine inhibitor of [Ca^2+^] L-channels, depolarization led to an increase in free cytosolic [Ca^2+^] concentration. [Ca^2+^]_i_ was significantly higher than under control conditions and appears to be similar to TMZ alone. Similar analysis was conducted with ER receptors. The TMZ induced [Ca^2+^]_i_ increase was completely abolished by xestospongin C, an antagonist of inositol trisphosphate receptor (IP3R). Ryanodine, an RyR-specific antagonist, did not significantly influence TMZ-induced [Ca^2+^]_i_ release (**Figure 3D**).

### Effects on Cell Proliferation by TMZ and/or [Ca^2+^]_i_-Related Inhibitors

**FIGURE 4:**
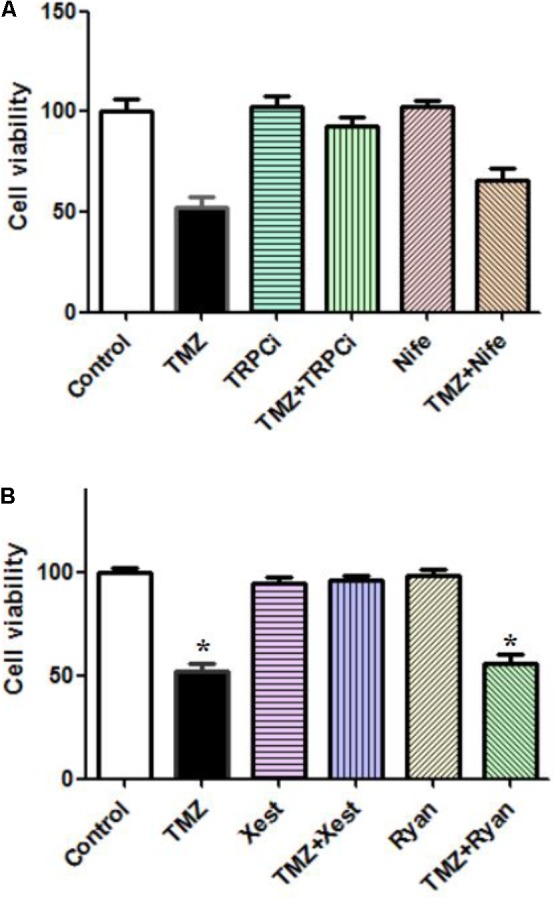
Effect of TRPC1 or IP3R inhibitors, combined with TMZ, on the proliferation of U251 cells. **(A)** Cells were incubated for 20 min and MTT analysis was performed in the absence of any drug (Control) or in the presence of 5 μM TMZ. In some experiments, 2 μg/mL TRPC1 neutralizing antibody (TRPCi) and 1 μM Nifedipine (Nife) were added 20 min before the addition of TMZ. **(B)** Cells were incubated for 20 min, and MTT analysis was performed in the absence of any drug (Control) or in the presence of 5 μM TMZ. In some experiments, 1 μM Xestspongin C (Xest) and 1 μM Ryanodine (Ryan) were added 20 min prior to the addition of TMZ. Data are presented as the mean ± SEM of six replicates. ^∗^Indicates a statistically significant (*P* < 0.05) difference from the negative control group.

The TMZ inhibition of cell proliferation was completely abolished by TRPC1 neutralizing antibody and xestospongin C, an antagonist of IP3R (**Figures 4A,B**). Similar to the effect on [Ca^2+^]_i_, the TMZ inhibition of cell proliferation was incompletely affected by nifedipine and ryanodine. These [Ca^2+^]_i_ and cell proliferation data suggest roles for TRPC1 and IP3R, although it is not clear what the precise roles of these molecules in TMZ-induced glycogenolysis might be.

### Effects on ERK_1/2_ Phosphorylation by Acute TMZ Treatment and/or Inhibitors of Signaling Pathways

**FIGURE 5:**
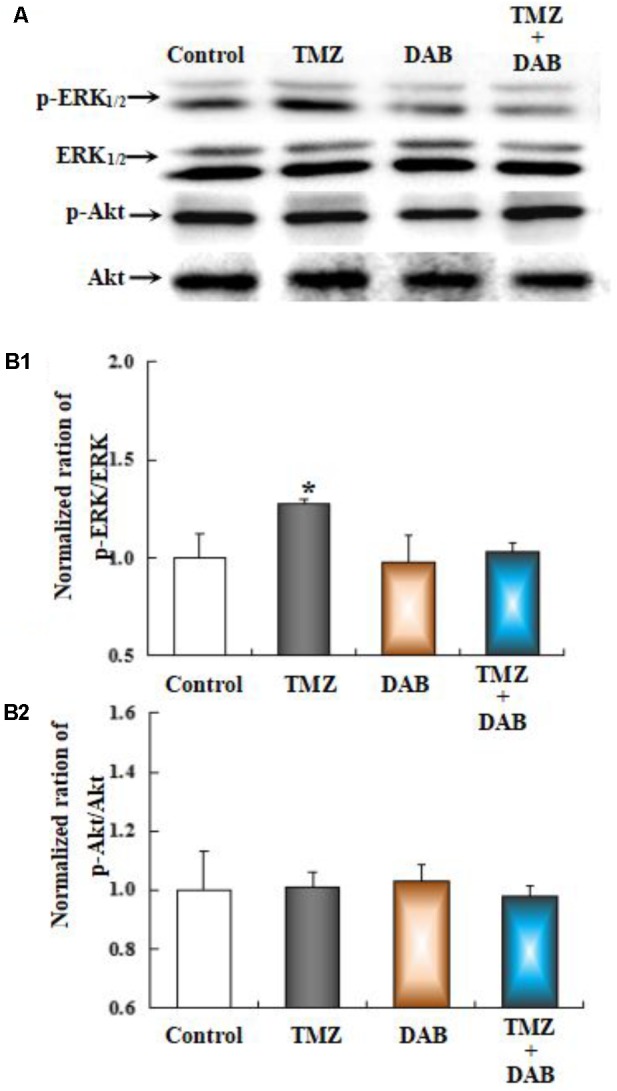
The upregulation of TMZ-induced ERK_1/2_ phosphorylation in U251 cells required glycogenolysis. **(A)** Cells were incubated for 20 min in the absence of any drug (Control) or in the presence of 5 μM TMZ. In some experiments, 10 mM DAB, an inhibitor of glycogenolysis, was added 4 h before the addition of TMZ. **(B1)** Mean ERK phosphorylation was quantified as ratios between p-ERK_1/2_ and ERK_1/2_. **(B2)** Average Akt phosphorylation was quantified as ratios between p-Akt and Akt. Similar results were obtained from three independent experiments. SEM values are indicated by vertical bars. ^∗^Indicates a statistically significant (*P* < 0.05) difference from the negative control group.

The effect of TMZ on ERK_1/2_ phosphorylation and AKT phosphorylation was studied in U251 glioma cells, after acute TMZ exposure for 20 min. As shown in **Figure 5A**, TMZ induced a statistically significant increase in ERK_1/2_ phosphorylation. The results are derived from three averaged experiments normalized to total ERK_1/2_, amounting to approximately 130% of control ERK_1/2_ phosphorylation. DAB, a GPBB and glycogenolysis inhibitor, rescued increased ERK_1/2_ phosphorylation. Scanning and averaging of the three plots showed the effect of TMZ on the phosphorylation of AKT. Total AKT protein was not significantly different from control cultures.

**FIGURE 6:**
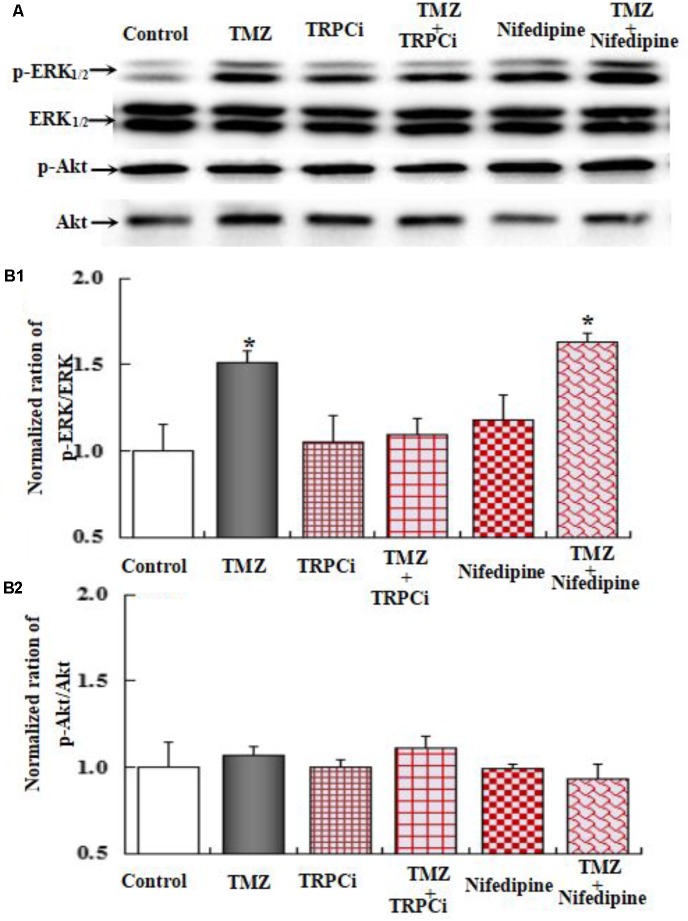
Upregulation of TMZ-induced ERK_1/2_ phosphorylation by TRPC1 in U251 cells. **(A)** Cells were incubated for 20 min in the absence of any drug (Control) or in the presence of 5 μM TMZ. In some experiments, 2 μg/mL TRPC1 neutralizing antibody (TRPCi) and 1 μM Nifedipine (Nife) were added 20 min before the addition of TMZ. **(B1)** Mean ERK phosphorylation was quantified as ratios between p-ERK_1/2_ and ERK_1/2_. **(B2)** Mean Akt phosphorylation was quantified as ratios between p-Akt and Akt. Similar results were obtained from three independent experiments. SEM values are indicated by vertical bars. ^∗^Indicates a statistically significant (*P* < 0.05) difference from the negative control group.

**FIGURE 7:**
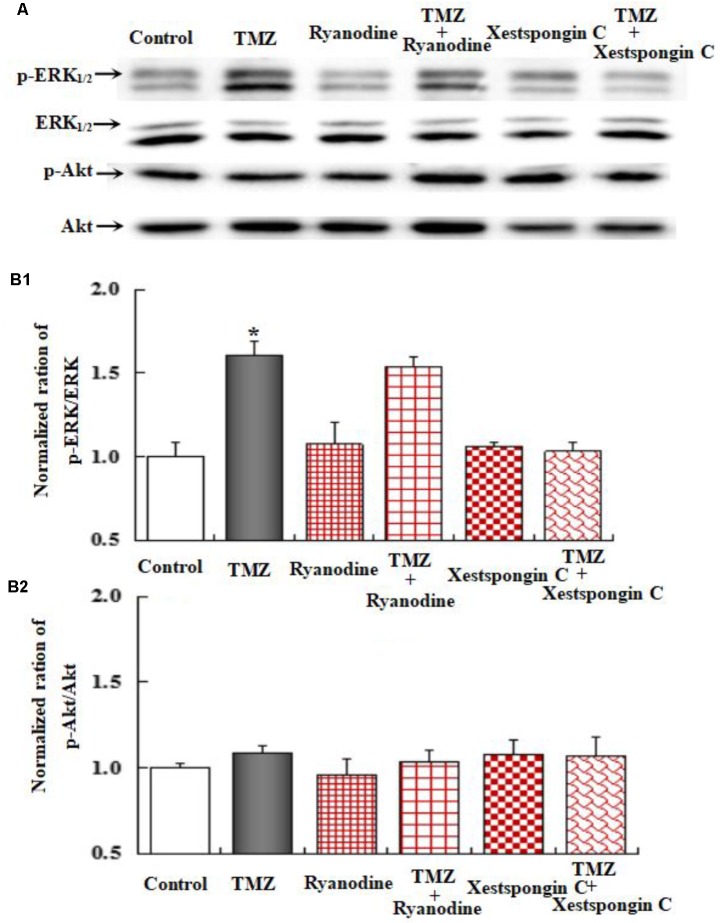
Upregulation of TMZ-induced ERK_1/2_ phosphorylation by IP3R in U251 cells. **(A)** Cells were incubated for 20 min in the absence of any drug (Control) or in the presence of 5 μM TMZ. In some experiments, 1 μM Xestspongin C (Xest) and 1 μM Ryanodine (Ryan) were added as in (**Figure 6**). **(B1)** Mean ERK phosphorylation was quantified as ratios between p-ERK_1/2_ and ERK_1/2_. **(B2)** Mean Akt phosphorylation was quantified as ratios between p-Akt and Akt. Similar results were obtained from three independent experiments. SEM values are indicated by vertical bars. ^∗^Indicates a statistically significant (*P* < 0.05) difference from the negative control group.

**FIGURE 8:**
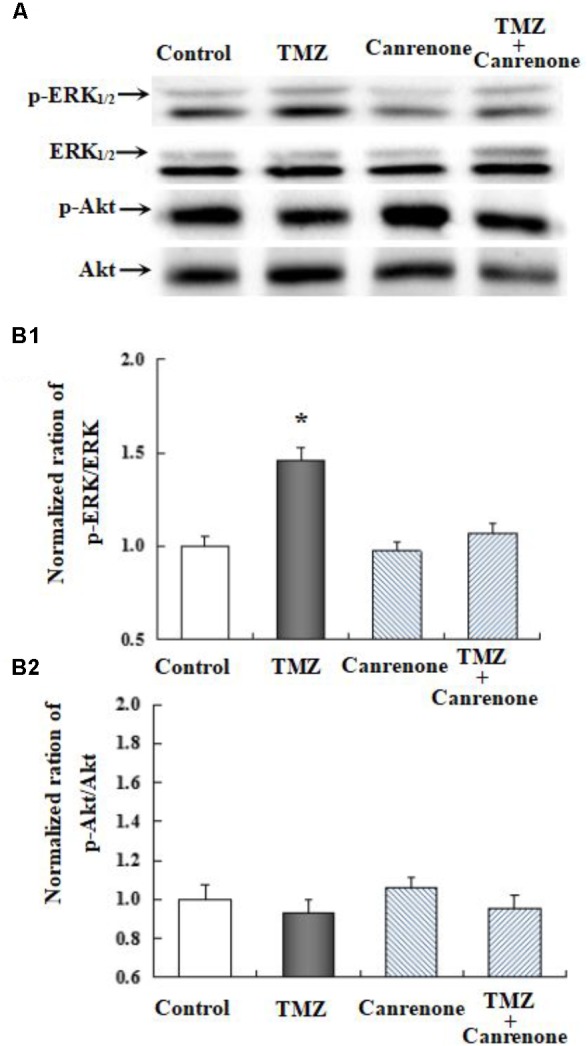
The upregulation of TMZ-induced ERK_1/2_ phosphorylation by Na,K-ATPase in U251 cells. **(A)** Cells were incubated for 20 min in the absence of any drug (Control) or in the presence of 5 μM TMZ. In some experiments, 10 μM Canrenone, an inhibitor of Na,K-ATPase, was added (**Figure 6**). **(B1)** Mean ERK phosphorylation was quantified as ratios between p-ERK_1/2_ and ERK_1/2_. **(B2)** Mean Akt phosphorylation was quantified as ratios between p-Akt and Akt. Similar results were obtained from three independent experiments. SEM values are indicated by vertical bars. ^∗^Indicates a statistically significant (*P* < 0.05) difference from the negative control group.

To further investigate the underlying mechanisms of TMZ-induced ERK_1/2_ phosphorylation, U251 cell cultures were treated with TRPC1 neutralizing antibody, nifedipine (a dihydropyridine inhibitor of L-channels for [Ca^2+^]_i_), xestospongin C (an IP_3_ receptor blocker), ryanodine (a RyR-specific antagonist), and canrenone (a Na,K-ATPase inhibitor). AKT phosphorylation was also assessed. **Figures 6**–**8** demonstrate that ERK_1/2_ phosphorylation was induced by TMZ and significantly inhibited by TRPC1 neutralizing antibody, xestospongin C, and canrenone, which on their own had no effect. Compared to TMZ-treated cells, no significant change in ERK_1/2_ phosphorylation was observed in cells treated with nifedipine or ryanodine (**Figures 6**, **7**). However, ERK_1/2_ phosphorylation was increased to a higher level when compared to control cell cultures. These data suggest that TMZ-induced ERK_1/2_ phosphorylation requires glycogenolysis and TRPC1/IP3R-induced [Ca^2+^]_i_ release.

### Effects on Na,K-ATPase and MGMT Protein Expression by Chronic TMZ Treatment and/or Inhibitors

**FIGURE 9:**
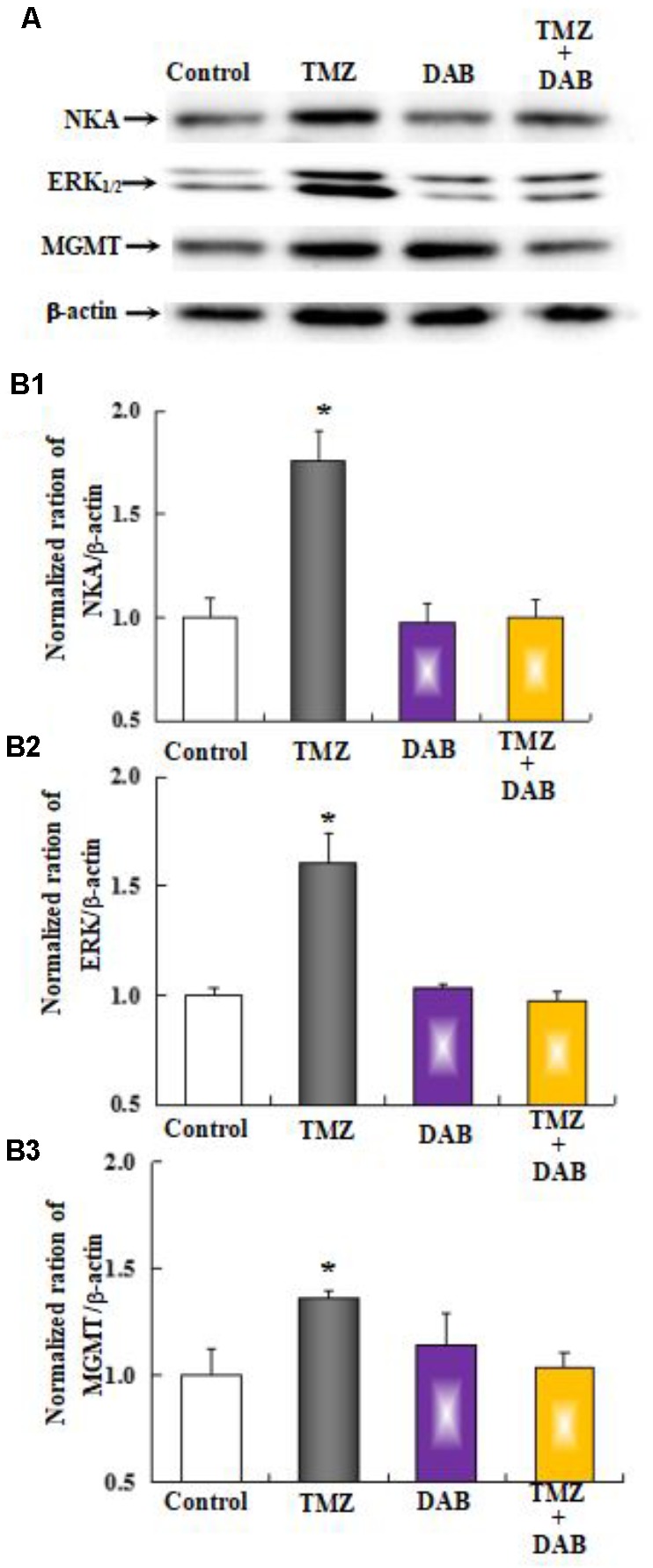
The upregulation of TMZ-induced Na,K-ATPase, ERK_1/2_, and MGMT expression requires glycogenolysis in U251 cells. **(A)** Cells were incubated for 48 h in the absence of any drug (Control) or in the presence of 5 μM TMZ. In some experiments, 10 mM DAB, an inhibitor of glycogenolysis, was added 4 h before the addition of TMZ. **(B1)** Mean Akt expression was quantified as ratios between Akt and β-actin. **(B2)** Mean ERK_1/2_ expression was quantified as ratios between ERK_1/2_ and β-actin. **(B3)** Mean MGMT expression was quantified as ratios between MGMT and β-actin. Similar results were obtained from three independent experiments. SEM values are indicated by vertical bars. ^∗^Indicates a statistically significant (*P* < 0.05) difference from the negative control group.

The effect of chronic TMZ on the MAPK signaling pathway, and on the expression of MGMT, a crucial target for TMZ in glioma cells, was explored. As shown in **Figure 9**, chronic treatment with TMZ for 48 h resulted in a significant increase (two- to threefold over control values) in the expression of Na,K-ATPase, ERK_1/2_, and MGMT proteins, normalized to the housekeeping protein β-actin. Pretreatment with DAB, a specific antagonist of GPBB and glycogenolysis, eliminated the TMZ effect on Na,K-ATPase, ERK_1/2_, and MGMT proteins.

**FIGURE 10:**
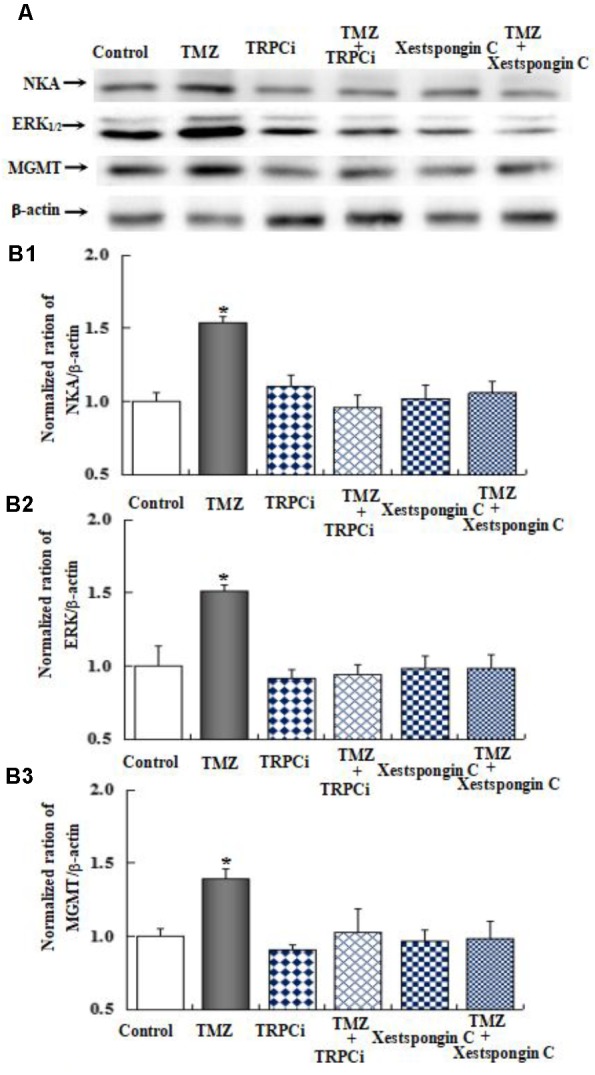
The upregulation of TMZ-induced Na,K-ATPase, ERK_1/2_, and MGMT expression by TRPC1 and IP3R in U251 cells. **(A)** Cells were incubated for 48 h in the absence of any drug (Control) or in the presence of 5 μM TMZ. In some experiments 2 μg/mL TRPC1 neutralizing antibody (TRPCi) and 1 μM Xestspongin C were added 4 h before the addition of TMZ. **(B1)** Mean Akt expression was quantified as ratios between Akt and β-actin. **(B2)** Mean ERK_1/2_ expression was quantified as ratios between ERK_1/2_ and β-actin. **(B3)** Mean MGMT expression was quantified as ratios between MGMT and β-actin. Similar results were obtained from three independent experiments. SEM values are indicated by vertical bars. ^∗^Indicates a statistically significant (*P* < 0.05) difference from the negative control group.

**FIGURE 11:**
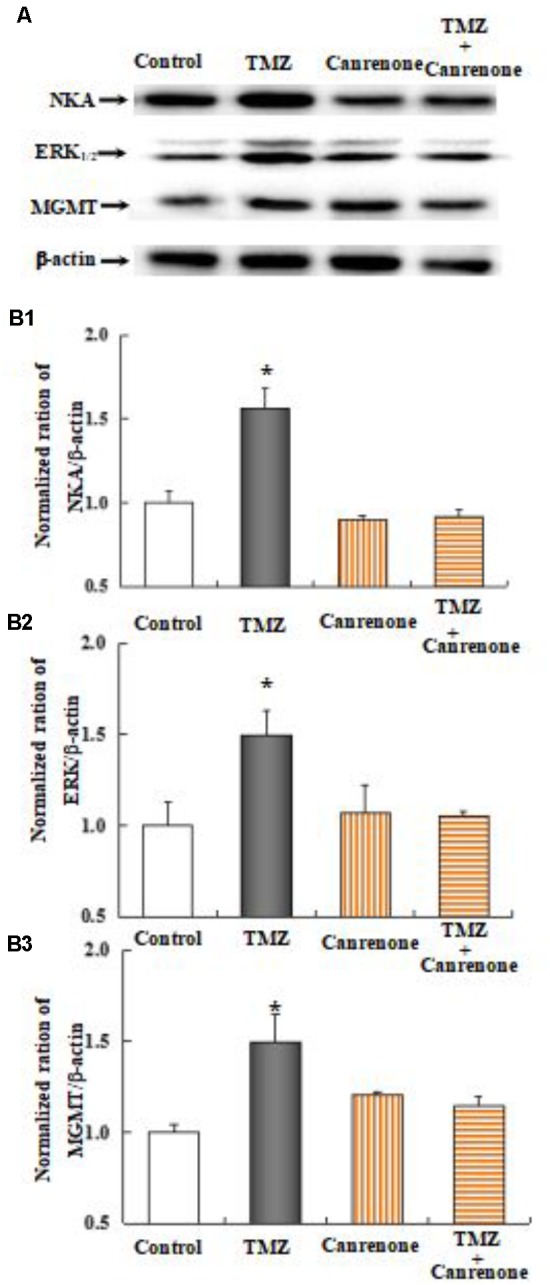
Upregulation of TMZ-induced ERK_1/2_ and MGMT expression by activation of Na,K-ATPase in U251 cells. **(A)** Cells were incubated for 48 h in the absence of any drug (Control) or in the presence of 5 μM TMZ. In some experiments 10 μM Canrenone, an inhibitor of Na,K-ATPase, was added 4 h before the addition of TMZ. **(B1)** Average mean expression was quantified as ratios between Akt and β-actin. **(B2)** Mean ERK_1/2_ expression was quantified as ratios between ERK_1/2_ and β-actin. **(B3)** Mean MGMT expression was quantified as ratios between MGMT and β-actin. Similar results were obtained from three independent experiments. SEM values are indicated by vertical bars. ^∗^Indicates a statistically significant (*P* < 0.05) difference from the negative control group.

**FIGURE 12:**
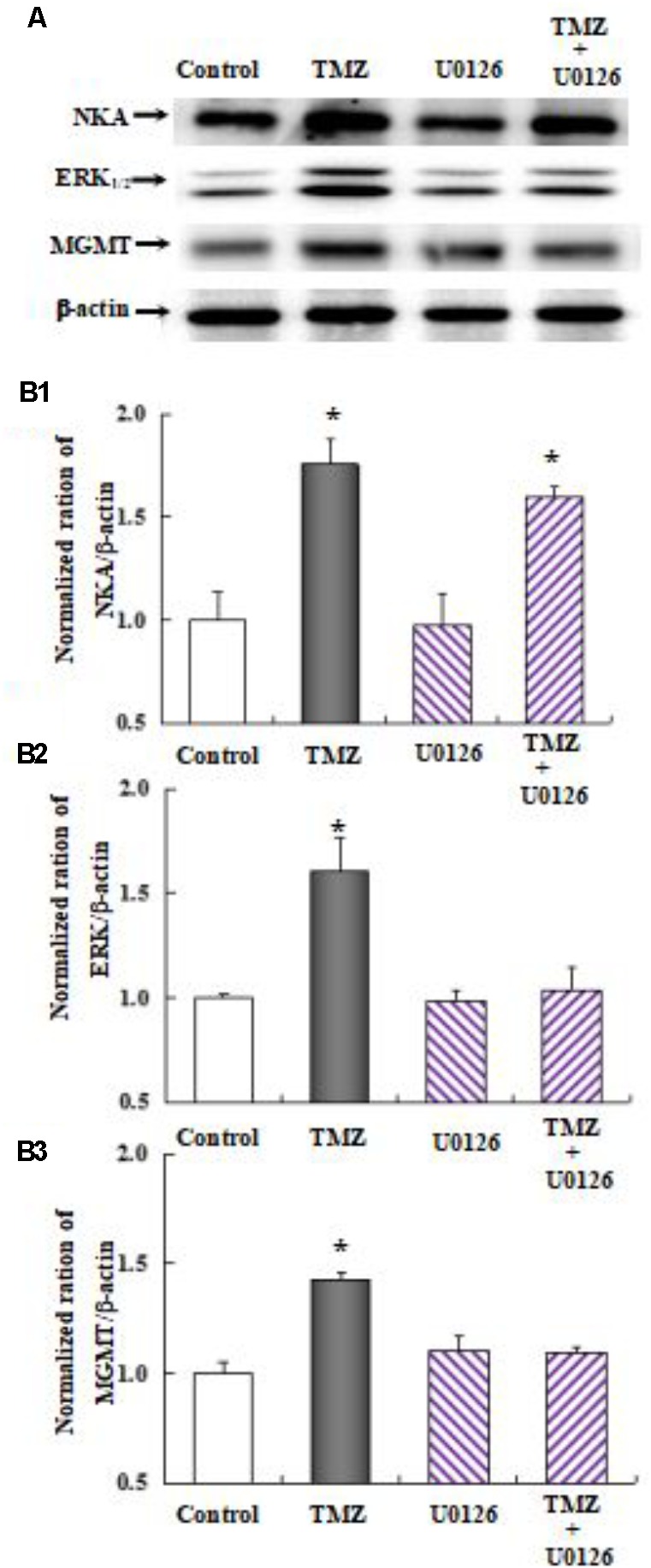
Upregulation of TMZ-induced MGMT expression by activation of ERK_1/2_ in U251 cells. **(A)** Cells were incubated for 48 h in the absence of any drug (Control) or in the presence of 5 μM TMZ. In some experiments 10 μM U0126, an inhibitor of MEK_1/2_, was added 4 h before the addition of TMZ. **(B1)** Mean Akt expression was quantified as ratios between Akt and β-actin. **(B2)** Mean ERK_1/2_ expression was quantified as ratios between ERK_1/2_ and β-actin. **(B3)** Mean MGMT expression was quantified as ratios between MGMT and β-actin. Similar results were obtained from three independent experiments. SEM values are indicated by vertical bars. ^∗^Indicates a statistically significant (*P* < 0.05) difference from the negative control group.

**FIGURE 13:**
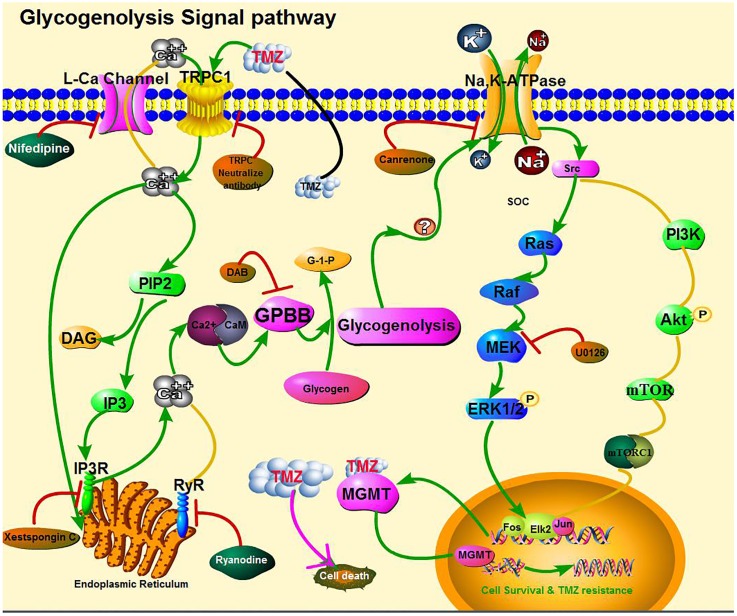
Cartoon illustrating signaling pathways activated by temozolomide (TMZ) in glioma cells. TMZ plays a dual role by both (1) inhibiting cell proliferation (pink solid line) and (2) inducing acquired TMZ resistance via the upregulation of MGMT. The latter effect requires [Ca^2+^]_i_-dependent glycogenolysis via the Na,K-ATPase/ERK_1/2_ signaling pathway (green solid lines). Activation of the second pathway leads to an increase in [Ca^2+^]_i_ by TRPC1 in the cell membrane and IP3R in the ER, which act upon the intracellular Ca^2+^-sensitive site of calmodulin (CaM) protein, possibly activating glycogen phosphorylase activation and the subsequent stimulation of glycogenolysis, necessary for pathway function. The ouabain signaling pathway is shown at the top of the figure. Na,K-ATPase was activated possibly by glycogenolysis and trans-activated by the MAPK pathway, which was confirmed by our own previous studies and our current inhibitor studies. Inhibitory effects on the upregulation of MGMT expression, after addition of TMZ, are shown in brown ovals. No effect of other inhibitors on [Ca^2+^]_i_ release and upregulation of Na,K-ATPase, ERK_1/2_, and MGMT are shown in green ovals.

As shown in **Figure 10**, the effect of TMZ chronic treatment on Na,K-ATPase, ERK_1/2_, and MGMT protein was abolished by TRPC1 neutralizing antibody and xestospongin C, an antagonist of IP3R. **Figure 11** demonstrates TMZ-mediated upregulation of Na,K-ATPase expression, which was confirmed by the inhibitory effect of canrenone, a specific antagonist of Na,K-ATPase, which was similar to the effect on ERK_1/2_ and MGMT expression. Furthermore, the upregulation of TMZ-induced ERK_1/2_ and MGMT expression was abolished by U0126, an inhibitor of ERK_1/2_, phosphorylation, with no effect on Na,K-ATPase expression, as shown in **Figure 12**. Taken together, these data indicate that the inhibition of downstream signaling did not inhibit upstream signaling. Furthermore, canrenone inhibited the activation of Na,K-ATPase before the transactivation of ERK_1/2_ and upregulation of MGMT. Overall, as shown in **Figure 13**, TMZ required [Ca^2+^]_i_-dependent glycogenolysis, which led to Na,K-ATPase/ERK_1/2_ phosphorylation and the upregulation of these proteins, as well as MGMT activation which induced TMZ-resistance in glioma cells.

## Discussion

This study reports, for the first time, that plasma GPBB concentrations differ between high-grade glioma patients with TMZ sensitivity and TMZ resistance. Our results demonstrate that [Ca^2+^]_i_-dependent glycogenolysis is required for TMZ resistance, stimulated by both the transactivation of Na,K-ATPase/ERK_1/2_ and the upregulation of MGMT expression in glioma cells. This is graphically illustrated in **Figure 13**, wherein TRPC1 and IP3R release [Ca^2+^]_i_ with activation of Na,K-ATPase and ERK_1/2_, the latter process was glycogenolysis-dependent and may upregulate the expression of MGMT protein.

Glycogen levels in the mammalian brain are generally reported to be ∼3 μmol/g wet weight and located almost exclusively in astrocytes (Ibrahim, 1975). In this study, the glycogen content of U251 glioma cells was found to be 80 nmol per mg protein, which corresponds to 16 mol/g wet weight. Glycogen phosphorylase isoenzyme BB (GPBB) is a key enzyme that degrades glycogen and is normally abundant in the brain and myocardium. GPBB converts inactive phosphorylase a into active phosphorylase b in the brain. The median plasma GPBB concentration in patients with high-grade glioma is almost two- to threefold greater than in non-malignant patients, with a cut-off value for the diagnosis of an acute coronary syndrome at 10 ng/ml (Lee et al., 2012). There is no evidence that patients with glioma have an increased risk for ischemic myocardial damage. It is therefore more likely that the increase in plasma GPBB is attributed to GPBB release from brain tumors and not the heart. Furthermore, GPBB released from a glioma and into the blood, with a non-intact blood-brain-barrier (BBB), would be a reasonable expectation.

Interestingly, these results demonstrate median plasma GPBB concentration to be significantly higher in high-grade glioma patients with TMZ resistance than in TMZ-sensitive patients. This result remained significant in an IDH 1 mutant cohort. The mutation of IDH1 in glioma is associated with a hyper-methylation phenotype, which activates HIF and inactivates α-ketoglutarate in glioma (Fu et al., 2012; Lu et al., 2012; Turcan et al., 2012). These findings suggest that the accumulation of 2-hydroxyglutarate and the activation of glycogenolysis, as a result of IDH mutation, keep glioma cells in an undifferentiated state and less sensitive to TMZ. These results also show that GPBB plasma concentrations are dramatically higher in patients with TMZ resistance than those with TMZ sensitivity, regardless of 1p/19q co-deletion or MGMT methylation status.

Increased glucose uptake and enhanced glycolytic rates are found in most malignancies, with abrogation of metabolism for tumor cell growth. Glycogen synthase kinase (GSK)3beta promotes glycogen synthase and the reduction of glycogenolysis in the brain and glioma cells. GSK3beta regulates differential responses to chemotherapy in ovarian tumor cells and glioblastoma cells (Luo, 2009; Pyko et al., 2013). The results herein indicate that chronic TMZ treatment induced a significant upregulation of MGMT protein that required glycogenolysis. MGMT promoter methylation inhibits the expression of MGMT protein and enhances tumor sensitivity to TMZ. Nevertheless, MGMT promoter methylation may be intrinsic to TMZ resistance, with the over expression of MGMT stimulated by signaling pathways as a potential means by which to acquire TMZ resistance. Interestingly, our results found that TMZ inhibits glioma cell proliferation, which was sufficiently pronounced to induce TMZ resistance via the upregulation of MGMT, which requires glycogenolysis.

Glycogen phosphorylation by GPBB shows an essentially identical requirement for an increase in free [Ca^2+^]_i_ concentration. The absolute [Ca^2+^]_i_ dependence of glycogenolysis was first demonstrated in muscle (Ozawa, 2011). Our present results are consistent with this earlier conclusion in that increased [Ca^2+^]_i_ and cell proliferation with TMZ were completely abolished by TRPC1 neutralizing antibody or xestospongin C, acting as an antagonist of IP3R, and incompletely abolished by ryanodine, acting as a RyR-specific antagonist. cAMP was sufficient to induce glycogenolysis and an increased glycogenolytic rate, but only when [Ca^2+^]_i_ was simultaneously increased (Ververken et al., 1982; Choi et al., 2012; da Rocha et al., 2014). However, Embi et al. (1980) suggested that the activity of gGSK-3 was unaffected by cAMP, [Ca^2+^]_i_ and the specific protein inhibitor of cAMP-dependent protein kinase. In the present study, we investigated the potential role of TMZ in the activation of glycogenolysis using cAMP to assess the signaling pathways leading to increased expression of MGMT. From our previous studies, we know that the addition of 5 mM K^+^ and ouabain stimulate a pathway via Na,K-ATPase, which acts downstream of MAPK. Although many second messengers are identical in this pathway, the IP3R receptor is an intermediate only in the ouabain-activated pathway. Furthermore, L-channels are only involved in the pathway when opened by a high K^+^ concentration. We therefore assessed the IP3R receptor inhibitor, xestospongin, which inhibits the expression of Na,K-ATPase, ERK_1/2_, and MGMT as well as the acute phosphorylation of Na,K-ATPase and ERK_1/2_ during glycogenolysis evoked by TMZ. A similar effect was inhibited by a component of store-operated [Ca^2+^] channels (SOCs), TRPC1 neutralizing antibody, and [Ca^2+^] entry, suggesting the participation of a pathway initiated by endogenous ouabains. This hypothesis was verified by canrenone inhibition (Yan et al., 2013; Liang et al., 2014; Song et al., 2015). However, both chronic and acute TMZ effects were unaffected by the L-channel inhibitor nifedipine and the RyR-specific antagonist ryanodine. TMZ increased [Ca^2+^] influx through TRPC1 and by activation of IP3R in the ER. Recent studies have also shown that SOC operation stimulates glycogenolysis (Müller, 2014; Mathieu et al., 2016).

The TMZ evoked an increase in MGMT expression with a glycogenolysis requirement for metabolic support of signaling processes. Chen et al. (2014) suggested, in agreement with ((s))Munoz et al. (2014) finding, that Na,K-ATPase may represent a therapeutic target for the treatment of glioblastoma by sensitizing glioblastoma cells to TMZ. Ouabain, acting as a representative Na,K-ATPase inhibitor, dramatically enhanced cell death in a TMZ-resistant cancer cell line (Clark et al., 2017). Several studies have also shown that ERK_1/2_ signaling is critical for TMZ glioma resistance via the TGF-beta 1-dependent activation of Smad/ERK signaling and via EGFR activation of the JNK-ERK_1/2_-AP-1 axis. Indeed, inhibition of Na,K-ATPase and ERK_1/2_ was documented and confirmed in this study to overcome acquired TMZ resistance by the same mechanism, as described previously (Tanabe et al., 2012; Riganti et al., 2013; Sun et al., 2013; Zeng et al., 2017). This is of special interest because targeting Na,K-ATPase and ERK_1/2_ may represent a novel strategy and improve glioma TMZ sensitivity.

There are some limitations to this study. First, blood samples from high-grade glioma patients were not available during treatment and hence could not be evaluated. Second, the predictive value during TMZ treatment could not be determined. Another limitation is that the mechanism underlying the activation of Na,K-ATPase and ERK_1/2_, and how they affect the expression of MGMT, remains unclear. We further focus on the potential mechanism of TMZ-affect [Ca^2+^]_i_ release and acquired alteration of MGMT expression by glycogenolysis. Finally, the predictive value of plasma GPBB concentrations needs to be verified in a larger cohort. In addition, short follow-up times for this study may result in survival data that are inaccurate.

## Conclusion

This study has shown that plasma GPBB concentration is a novel biomarker that can be used to predict the response to TMZ in high-grade glioma patients. These findings suggest that TMZ inhibits cell proliferation and induces acquired TMZ resistance by upregulating MGMT. The latter process requires [Ca^2+^]_i_-dependent glycogenolysis via the Na,K-ATPase/ERK_1/2_ signaling pathway.

## Author Contributions

JX performed the literature database search and wrote the manuscript. JX and YZ performed the experiments. YZ, XG, and TS contributed to the vital insights and proofread the manuscript. JX and TS conceptualized the research topic.

## Conflict of Interest Statement

The authors declare that the research was conducted in the absence of any commercial or financial relationships that could be construed as a potential conflict of interest.
